# Trop2 is overexpressed in gastric cancer and predicts poor prognosis

**DOI:** 10.18632/oncotarget.6733

**Published:** 2015-12-22

**Authors:** Wei Zhao, Huijun Zhu, Shu Zhang, Hongmei Yong, Wei Wang, Yan Zhou, Bing Wang, Jinbo Wen, Zhenning Qiu, Guipeng Ding, Zhenqing Feng, Jin Zhu

**Affiliations:** ^1^ Department of Pathology, Nanjing Medical University, Nanjing 210029, China; ^2^ School of Public Health, Nantong University, Nantong 226019, China; ^3^ Department of Pathology, Affiliated Hospital of Nantong University, Nantong 226019, China; ^4^ Department of Oncology, Huai'an Hospital Affiliated of Xuzhou Medical College and Huai'an Second People's Hospital, Huai'an 223001, China; ^5^ Department of Oncology, AoYoung Hospital, Zhangjiagang, Jiangsu 215617, China; ^6^ Department of Epidemiology, School of Public Health, Nanjing Medical University, Nanjing 210029, China; ^7^ Key Laboratory of Antibody Technique of Ministry of Health, Nanjing Medical University, Nanjing 210029, China; ^8^ Jiangsu Collaborative Innovation Center for Cancer Personalized Medicine, Nanjing Medical University, Nanjing 210029, China; ^9^ Key Laboratory of Cancer Biomarkers, Prevent and Treatment, Cancer Center, Nanjing Medical University, Nanjing 210029, China; ^10^ Huadong Medical Institute of Biotechniques, Nanjing 210029, China

**Keywords:** Trop2, prognosis, gastric cancer

## Abstract

The cell surface protein Trop2 is overexpressed in a variety of human cancers. Trop2 expression increases tumor development and metastasis and reduces patient survival. However, little is known about the role of Trop2 expression and its prognostic value in gastric cancer (GC), particularly in Chinese populations. We analyzed Trop2 expression in GC tissues collected from Chinese GC patients. Quantitative real-time polymerase chain reaction (qRT-PCR) and immunohistochemistry on tissue microarrays were performed to assess levels of Trop2 mRNA and protein in GC, and correlations between Trop2 expression and clinical characteristics and prognosis were analyzed. Trop2 expression was higher in GC tissues than in neighboring non-tumor tissues. Increased Trop2 protein levels in GC were associated with increased differentiation, tumor node metastasis stage, tumor size, lymph node metastasis, distant metastasis, and *H. pylori* infection. GC patients with high Trop2 expression also had poor overall survival rates. These data suggest Trop2 is a useful prognostic biomarker for GC.

## INTRODUCTION

Gastric cancer (GC) is a leading cause of global cancer-related mortality [1]. GC prevalence is highest in Asia, and the majority of GC patients are diagnosed at an advanced stage [2–3]. Due to the high rates of metastasis and recurrence, the prognosis of GC patients is poor: 5-year survival rates are less than 20% [4]. *Helicobacter pylori* (*H. pylori*) infection and intestinal metaplasia (IM) are common complications in gastric cancer patients [5–8]. Identifying new genetic markers to help diagnose GC earlier will help to improve cure rates and reduce complications, including *H. pylori* and other chronic gastric infections.

The human trophoblast cell surface (TACSTD2/Trop2/M1S1/GA733–1) gene is located at chromosome 1q32 [9]. Trop2 is a 36-kDa single-pass transmembrane protein expressed primarily in epithelial cells [10] and was first identified in human trophoblasts [11]. Trop2 has several binding partners, including Claudin 1, Claudin 7, Cyclin D1, protein kinase C (PKC), PIP2, and insulin-like growth factor 1 (IGF-1) [11, 32–33]. By binding to these targets, Trop2 affects tight junctions at the epithelial barrier [26]; increases tumor proliferation [34], podosome formation, and Raf and NF-kappa activation [11, 26]; and suppresses IGF-1R signaling [35]. Trop2 is overexpressed in various epithelial tumors [12–15], and its expression correlates with aggressive tumor behavior [16]. In contrast to tumor cells, somatic adult tissues show little or no Trop2 expression [11, 17].

Trop2 increases tumor recurrence, progression, and invasiveness [18–21], and has been used as a prognostic marker in several types of cancer [22]. It is overexpressed in oral squamous epithelial cell carcinoma [13], breast cancer [18], colon cancer [23], pancreatic cancer [24], and cervical cancer [25]. It is also upregulated in certain malignant hematological diseases, including Non-Hodgkin's lymphoma (NHL) and leukemia [26]. Antibodies such as human anti-Trop2 antibody IgG and fragment Fab, which reduce Trop2 expression, also inhibit cell proliferation, induce apoptosis, and delay migration in some cancer cell types, both *in vitro* and *in vivo* [27–31].

The role of Trop2 in GC is less well understood. Muhlmann et al. detected elevated Trop2 expression in a group of 104 Austrian patients with intestinal-type carcinomas [14]. Because the incidence and lethality of GC differs between populations, it would seem useful to explore Trop2 expression levels in both benign and malignant gastric tissues from different patient groups. In this study, we analyzed Trop2 expression in primary GCs from 600 Chinese patients and compared it with expression in matched neighboring non-tumor tissues. We also examined the relationship between Trop2 expression, clinical characteristics, and overall survival in GC.

## RESULTS

### Trop2 mRNA was overexpressed in GC tissues

**Figure 1:**
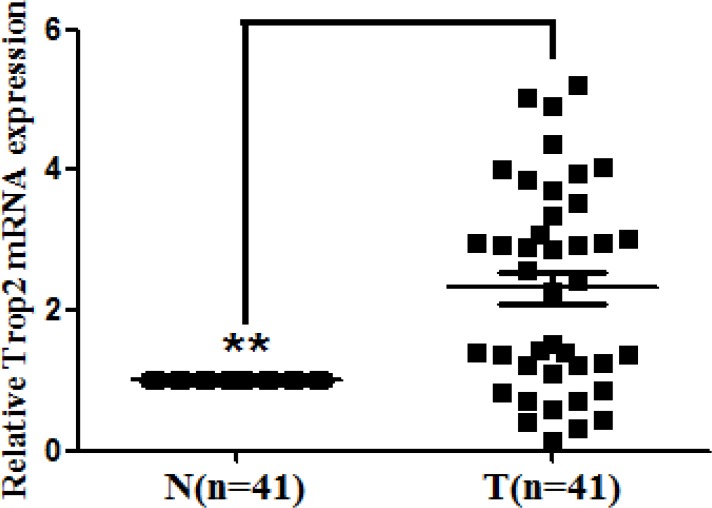
Trop2 mRNA expression in 41 GC tissue pairs Trop2 mRNA expression was examined by qRT-PCR and normalized to β-actin. Trop2 mRNA levels were higher in the 41 GC tissues (T) than in matched tumor neighbor tissues (N) (**p < 0.001).

To investigate the expression of Trop2 mRNA, we performed qRT-PCR in 41 pairs of GC tissue and matched tumor neighbor tissue. Trop2 mRNA expression was 2.32 **±** 1.44 fold higher in GC tissues than in matched tumor neighbor tissues (*p* < 0.001, Figure 1).

### Trop2 protein was overexpressed in GC tissues

**Figure 2:**
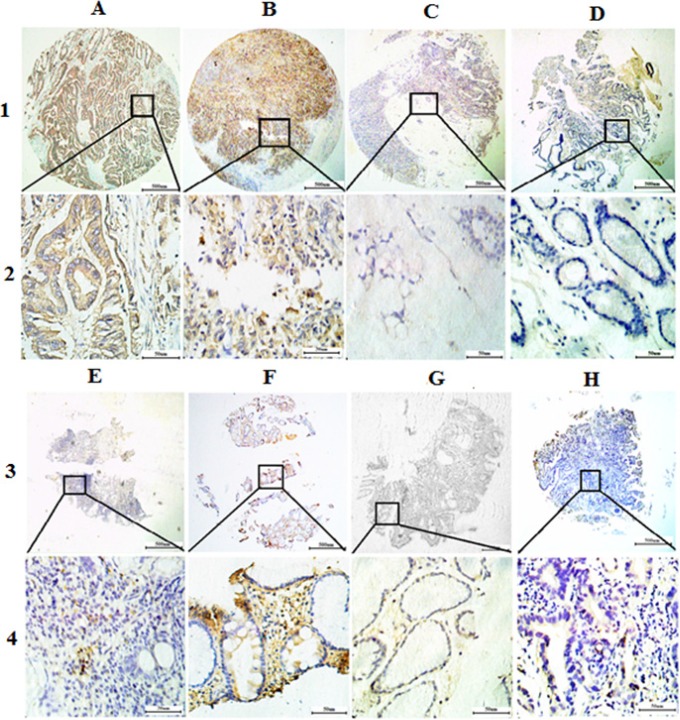
Representative images of Trop2 protein expression in gastric tissue TMA sections (**A**) Middle differentiated gastric cancer with high Trop2 expression (IHC score, 300); (**B**) Poor differentiated gastric cancer with high Trop2 expression (IHC score, 270); (**C**) Signet ring cell gastric cancer with low Trop2 expression (IHC score, 60); (**D**) Matched tumor neighbor tissues with low Trop2 expression (IHC score, 30); (**E**) Chronic gastritis with low Trop2 expression (IHC score, 30); (**F**) Intestinal metaplasia with high Trop2 expression (IHC score, 260); (**G**) Low-grade intraepithelial neoplasia with low Trop2 expression (IHC score, 30); (**H**) High-grade intraepithelial neoplasia with low Trop2 expression (IHC score, 60); Row 1 and 3 are Trop2 staining with × 4 (bar = 500 μm), Row 2 and 4 are Trop2 staining with × 40 (bar = 50 μm).

Trop2 protein, which was localized to the membrane and cytoplasm using immunohistochemistry, was overexpressed in GC tissues (Figure 2). Using the x-tile software program for TMA data analysis (http://www.tissuearray.org/rimmlab), we defined high and low Trop2 expression levels based on overall survival in GC patients. A cutoff value of 130 cells was selected: counts between 0 and 130 were considered low expression, while counts of 131 to 300 were considered high expression. All Trop2 protein levels were classified as “Low” or “High” using these cutoff values prior to analysis.

High Trop2 expression was detected more often in gastric cancer tissues (66.30%, 398/600) than in matched tumor neighbor tissues (43.8%, 39/89) (Table 1). However, in Signet ring cell carcinoma, high Trop2 expression was only present in 43.5% of tissues (10/23). Interestingly, high Trop2 expression was most prevalent in intestinal metaplasia (IM) tissues (80.00%, 24/30). Low Trop2 expression levels were found less frequently in other gastric tissue types, including chronic gastritis (12.5%, 4/32), low-grade intraepithelial neoplasia (29.0%, 9/31), and high-grade intraepithelial neoplasia (37.5%, 18/48).

**Table 1:** Trop2 expression in gastric tissues

Characteristic	*n*	Trop2 expression (%)		χ^2^	*p*
		Low or no	High		
Stomach				76.705	< 0.001*
Cancer	600	202 (33.70)	398 (66.30)		
Matched tumor neighbor	89	50 (56.2)	39 (43.8)	32.55	< 0.001*
Chronic gastritis	32	28 (87.50)	4 (12.50)	11.65	< 0.001*
Intestinal metaplasia	30	6 (20.00)	24 (80.00)	9.90	0.084
Low-grade intraepithelial neoplasia	31	22 (71.0)	9 (29.0)	17.912	< 0.001*
High-grade intraepithelial neoplasia	48	30 (62.50)	18 (37.50)	16.934	< 0.001*

χ^2^ and *p* values for stomach overall includes all types gastric tissues. The χ^2^ and *p* values for matched tumor neighbor, chronic gastritis, intestinal metaplasia, low-grade intraepithelial neoplasia, and high-grade intraepithelial neoplasia are in comparison to the cancer group.

### Trop2 overexpression was associated with more advanced clinical characteristics in GC patients

We next investigated the relationship between Trop2 protein levels and patient clinical parameters (Table 2). High expression of Trop2 in GC was associated with increased differentiation (χ^2^ = 9.192, *p* = 0.027), Tumor Node Metastasis (TNM) stage (χ^2^ = 38.939, *p* < 0.001), tumor size (χ^2^ = 35.576, *p* < 0.001), lymph node metastases (χ^2^ = 17.638, *p* < 0.001), distant metastases (χ^2^ = 9.728, *p =* 0.001), and *H. pylori* infection (χ^2^ = 7.549, *p =* 0.005). However, there were no significant associations between Trop2 levels and gender, age, or histology type. (Table 2).

**Table 2:** Associations between high Trop2 expression and clinicopathologic characteristics in GC patients

Characteristic	*n*	Trop2 expression (%)		Pearson χ^2^	*p*
		Low or no	High		
Total	600	202 (34.70)	398 (66.30)		
**Gender**				0.557	0.504
Male	428	148 (34.60)	280 (65.40)		
Female	172	54 (31.40)	118 (68.60)		
**Age**				2.158	0.084
< 60	334	104 (31.10)	230 (68.90)		
≥ 60	226	98 (36.80)	168 (63.20)		
**Histological type**				9.187	0.057
Tubular	524	176 (33.60)	348 (66.40)		
Mixed (tubular and mucinous)	7	2 (28.60)	5 (71.40)		
Mucinous	34	10 (29.40)	24 (70.60)		
Signet ring cell	23	13 (56.50)	10 (43.50)		
Others^a^	12	1 (8.30)	11 (91.70)		
**Differentiation**				9.192	0.027*
Well	57	28 (49.10)	29 (50.90)		
Moderate	145	52 (35.90)	93 (64.10)		
Poor	329	97 (29.50)	232 (70.50)		
Others^b^	69	25 (36.20)	44 (63.80)		
**TNM stage**				38.939	< 0.001*
0	18	10 (50.60)	8 (44.40)		
Ia + Ib	111	60 (54.10)	51 (45.90)		
Ia + IIb	208	72 (34.60)	136 (65.40)		
IIIa+ IIIb	210	50 (23.80)	160 (76.20)		
IIIc + IV	53	10 (18.90)	43 (81.10)		
**Tumor size**				35.576	< 0.001*
T0	18	10 (55.60)	8 (44.40)		
T1a+T1b+T2	180	89 (49.40)	91 (50.60)		
T3+T4a+T4b	402	103 (25.60)	299 (74.40)		
**Lymph node metastases**				17.638	< 0.001*
N0	227	100 (44.10)	127 (55.90)		
N1	373	102 (27.30)	271 (72.70)		
**Distant metastases**				9.728	0.001*
M0	562	198 (35.20)	364 (64.80)		
M1	38	4 (10.50)	34 (89.50)		
***H. pylori* infection**				7.549	0.005*
Positive	475	147 (30.9%)	328 (69.1%)		
Negative	125	55 (44.0%)	70 (56.0%)		

a others include: pallipary adenocarcinoma, 5 cases; adeno-squamous carcinoma, 5 cases; squamous cell carcinoma, 3 cases; undifferentiated carcinoma, 2 cases; and neuroendocrine carcinoma, 1 case.

b others include everything besides tubular and papillary adenocarcinoma.

* *p* < 0.05.

### Trop2 protein overexpression was associated with poor prognosis in GC

**Figure 3:**
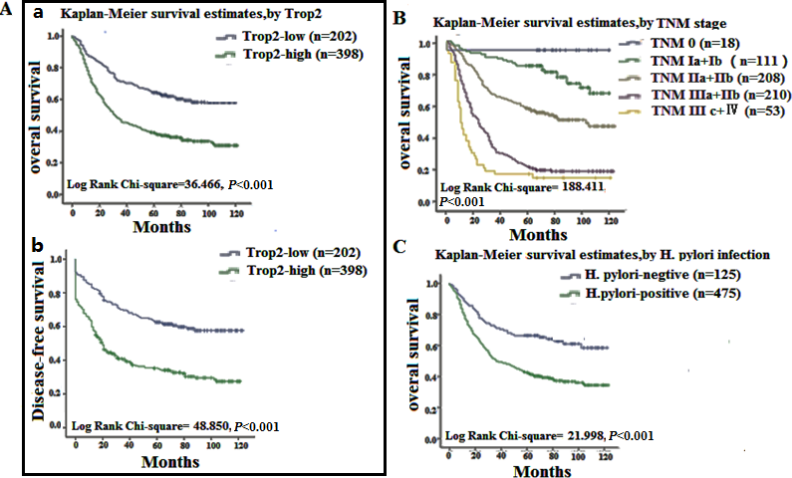
Survival curves for gastric cancer using the Kaplan-Meier method and the log-rank test (**A**); a Overall survival curves for patients with low or no Trop2 expression (blue line, 1) and patients with high Trop2 expression of (green line, 2); b disease-free survival curves for patients with low or no Trop2 expression (blue line, 1) and high Trop2 expression (green line, 2). (**B**) Overall survival curves by TNM stage; TNM 0 (blue line, 1), TNM Ia and Ib (green line, 2), TNM IIa and IIb (grey line, 3), TNM IIIa and IIIb (purple line, 4), TNM IIIc and IV (yellow line, 5). (**C**) overall survival curves for *H. pylori* negative (blue line, 1) and positive (green line, 2) patients.

We also investigated the relationship between Trop 2 expression and prognostic factors in GC using both univariate and multivariate analyses. High Trop2 expression (HR, 2.474, 95% CI, 1.765–3.468; *p* < 0.001) was associated with poor overall survival in the univariate analysis, and with increases in other prognostic markers, including TNM stage (HR, 2.245, 95% CI, 1.870–2.696; *p* < 0.001) and *H. pylori* infection (HR, 2.696, 95% CI, 1.795–4.008; *p* < 0.001) (Table 3). In the multivariate analysis, high Trop2 expression was associated with poor overall survival (HR, 1.819, 95% CI, 1.213–2.728; *p =* 0.004) and increases in TNM stage (HR, 2.265, 95% CI, 1.512–3.393; *p* < 0.001) and *H. pylori* infection (HR, 2.512, 95% CI, 1.575–4.006; *p* < 0.001) (Table 3). Kaplan-Meier survival curves showed that Trop2 overexpression was significantly associated with poor overall survival, as well as with TNM stage and *H. pylori* infection. Additionally, high Trop2 expression was associated with lower disease-free survival rates in GC patients (Figure 3). These results suggest that Trop2 expression, TNM stage, and *H. pylori* infection are prognostic markers in GC.

**Table 3:** Univariate and multivariate analysis of prognostic markers for overall survival in gastric cancer

	Univariate analysis			Multivariate analysis		
	HR	*p* > |z|	95%CI	HR	*p* > |z|	95%CI
**Trop2 expression**						
High vs Low or no	2.474	0.000*	1.765–3.468	1.819	0.004*	1.213–2.728
**Age**						
≤ 60 vs ≥ 60	0.733	0.057	0.532–1.009	–	–	–
**Gender**						
Male vs Female	1.083	0.658	0.761–1.541	–	–	–
**Histological type**						
Tubular vs Mixed (tubular and mucinous) vs Mucinous vs signet ring cells vs others^a^	0.924	0.388	0.773–1.105	–	–	–
**Differentiation**						
Well vs Moderate vs Poor	1.168	0.115	0.963–1.416	–	–	–
**TNM stage**						
0 vs Ia + Ib vs IIa + IIb vs IIIa + IIIb vs IIIc + IV	2.245	< 0.001*	1.870–2.696	2.265	< 0.001*	1.512–3.393
**Tumor size**						
Tis vs T1 vs T2 vs T3 vs T4	2.320	< 0.001*	1.758–3.062	–	–	–
**Lymph node metastases**						
N0 vs N1 vs N2 vs N3	3.657	< 0.001*	2.611–5.123	–	–	–
**Distant metastases**						
M0 vs M1	6.614	< 0.001*	2.317–18.878	–	–	–
**H. pylori infection**						
Positive vs Negative	2.696	< 0.001*	1.795–4.008	2.512	< 0.001*	1.575–4.006

a others include: pallipary adenocarcinoma, 5 cases; adeno-squamous carcinoma, 5 cases; squamous cell carcinoma, 3 cases; undifferentiated carcinoma, 2 cases; and neuroendocrine carcinoma, 1 case.

* *p* < 0.05.

## DISCUSSION

In this study, we used qRT-PCR and immunohistochemistry to examine Trop2 mRNA and protein levels in GC tissues from Chinese patients. Trop2 mRNA levels were much higher in GC tissues than in matched neighboring non-tumor tissues, as is the case in other tumors [34]. However, the relationship between mRNA and protein levels is not strictly linear and involves spatial-temporal disparities. To confirm that protein levels were also elevated in GC, we examined a microarray that included 830 samples of gastric tissue, 600 of which were GC, and the associated clinical and follow-up data. Trop2 protein was localized to the cell membrane and cytoplasm. As was the case with mRNA, high Trop2 protein expression was detected in a larger proportion of cancerous tissues (66.30%) than in neighboring non-cancerous tissues (43.8%). Furthermore, Trop2 overexpression predicted poor overall and disease-free survival. This is similar to the findings of Muhlmann et al., who reported that increased Trop2 expression accompanied poor disease-free and overall survival rates in patients with advanced intestinal-type carcinoma [14].

Although Muhlmann and colleagues also studied the relationship between Trop2 expression and GC, they used the Lauren classification, which only distinguishes between intestinal-type and diffuse-type cancers [14]. Here, we divided the 600 GC cases into several types according to the latest WHO classification guidelines [36]: tubular, mixed (tubular and mucinous), mucinous, Signet ring cell, and other (papillary adenocarcinoma, adeno-squamous carcinoma, squamous cell carcinoma, undifferentiated carcinoma, and neuroendocrine carcinoma). We found that Trop2 was overexpressed in all GC tissues with the exception of Signet ring cells. However, future studies examining higher numbers of each sample type could help clarify the impact of Trop2 in GC. Additionally, the molecular characteristics and epidemiology of GC vary between eastern and western countries [37–38]. The incidence of different types of GC has also changed over time [39]. Therefore, identifying the molecular characteristics of the various GC types in different populations could potentially increase the efficacy of targeted therapies.

GC is the most prevalent digestive system malignancy in the world, and China has more GC patients than any other country. Moreover, 40%–80% of the population in China is *H. pylori*-positive, but only a small proportion of infected patients develop GC [40]. Several reports have suggested that *H. pylori* infection can induce genetic and signaling pathway changes [5]. We found here that *H. pylori* infection was associated with high Trop2 expression and poor overall survival. It is possible that *H. pylori* infection may induce Trop2 overexpression, but the mechanism behind this induction needs to be explored in future studies.

Interestingly, our data also show that Trop2 may be more frequently overexpressed in intestinal metaplasia (IM) (80%) than in GC tissues (66.30%). This difference did not reach statistical significance, perhaps due to the small number of IM cases examined (*n =* 30 vs *n =* 600 for GC). Gastric mucosa IM lesions can develop into GC (intestinal-type) [41], and chronic gastritis can progress through AG, IM, and dysplasia to eventually result in GC [42]. However, Trop2 expression was elevated in only 29.03% of low-grade and 37.50% of high-grade intra-epithelial neoplasia tissues. Further studies will be necessary to determine the role of Trop2 in the progression from IM to GC. However, our results suggest that high Trop2 expression is related to poor prognosis in GC, and monitoring this biomarker may aid in the treatment of GC.

## METHODS

### Human tissue specimens and patient clinical information

A total of 830 formalin-fixed, paraffin-embedded (FFPE) stomach tissue samples were collected from 741 patients. This included 600 cancer tissues with 89 matched tumor neighbor tissues, and 32 chronic gastritis, 30 intestinal metaplasia, 31 low-grade intraepithelial neoplasia, and 48 high-grade intraepithelial neoplasia tissues. All tissue blocks were received from the Department of Pathology at the Affiliated Hospital of Nantong University between 2003 and 2010. Medical records for tissue donor patients included information on age, sex, Tumor Node Metastasis (TNM) stage, histological type, differentiation grade, and *H. pylori* infection. No patients received treatment (radiation therapy, chemotherapy, or immunotherapy) before surgical resection. Overall survival (OS) was defied as the period from initial diagnosis via biopsy to death. Information on patients who were alive at the last follow-up date were deleted from the analysis. Disease-free survival (DFS) was defied as the period from follow-up to recurrence. Forty-one additional freshly frozen gastric cancer and matching tumor neighbor tissues were obtained primarily from the First Affiliated Hospital of Nanjing Medical University. Tissues from Huai'an Second People's Hospital, Zhangjiagang AoYoung Hospital, and Affiliated Hospital of Nantong University were also included in this study. The study protocol was approved by the Human Research Ethics Committees of these hospitals.

### Tissue microarrays (TMA) and immunohistochemistry analysis (IHC)

The TMAs were generated in the Department of Pathology, Nantong University Affiliated Hospital, Nantong, Jiangsu, China, using the Tissue Microarrayer System Quick Ray (UT06, UNITMA, Korea) manual. Specifically, core tissue biopsies (2 mm in diameter) were taken from 70 individuals. FFPE blocks were made and then arranged in new recipient paraffin blocks. A total of 13 gastric TMAs were made. Four-micron sections were cut and placed on super frost-charged glass microscope slides to generate TMA slides. Tissue sections were deparaffinized and rehydrated through graded alcohols. Endogenous peroxidase activity was blocked by incubation in 3% H_2_O_2_. Tissues were placed in 0.01 M citrate buffer, pH 6.0, and heated in a microwave for antigen retrieval. Trop2 was detected using a polyclonal goat anti-human Trop2 antibody (dilution 1:200) (R & D, AF650). Reactions were detected with an Envision^™^ peroxidase kit (Dako, Carpinteria, CA, USA). Tissues were then incubated in 3, 3′-diaminobenzidine plus (Dako, Carpinteria, CA, USA), counterstained with Hematoxylin, dehydrated through graded alcohols, cleared in xylene, and coverslipped with permanent mounting media. Staining was quantified in all tissues without knowledge of clinical characteristics. Trop2 expression was scored using the semi-quantitative H-score method, which takes into account both the staining intensity and the percentage of cells at that intensity [43–44]. The following staining intensity scores were used: 0 indicated no staining; 1+ indicated weak staining; 2+ indicated moderate staining; and 3+ indicated intense staining. The total number of cells at each intensity level was multiplied by the corresponding intensity score to yield an intensity percentage score. Final staining scores were then calculated by summing the four intensity percentage scores; the minimum possible final staining score was 0 (no staining), and maximum possible score was 300 (100% of cells with 3+ staining intensity).

### Quantitative real-time polymerase chain reaction (qRT-PCR)

qRT-PCR was performed to determine Trop2 mRNA expression levels in 41 pairs of human GC tissue and matched tumor neighbor tissue. Tissue samples were snap-frozen in liquid nitrogen and stored at −80°C before RNA extraction. Total RNA was extracted from frozen samples using Trizol reagent (Invitrogen, Carlsbad, CA, USA) and reverse transcribed into cDNA using a PrimeScript^™^ RT reagent kit (Takara, Glen Burnie, MD) in accordance with the manufacturer's instructions. Human β-actin served as the internal control for determining Trop2 mRNA levels. The following primers were used for PCR reactions: human β-actin forward, 5′-TGGAGAAAATCTGGCACCAC-3′, and reverse, 5′-GATGATGCCTCGTTCTAC-3′, and Trop2 forward, 5′-TGTCCTGATGTGATATGTCTGAG-3′, and reverse, 5′-GGGTGAGAGTGGGTTGGG-3′ (Genescript. Nanjing, China). qPT-PCR was performed on an ABI PRISM 7500HT Sequence Detection System (Applied Biosystems, Foster City, CA, USA) in 96-well plates. The final volume for each reaction was 20 μL, which included 2 μL of cDNA template (corresponding to ∼40 ng of retro-transcribed total RNA), the primers (20 nmol/L each) and 2 × SYBR Green PCR Master Mixture (10 μL; Applied Biosystems). Cycle conditions were as follows: after an initial 2-minute hold at 50°C to allow AmpErase-UNG activity and 10 min at 95°C, the samples were cycled 40 times at 95°C for 15 seconds and 58°C for 1 minute. All experiments were performed in triplicate. Results were normalized to respective internal controls. The Ct-value for each sample was calculated using the ΔΔCt method [45–48], and results were expressed as 2^−ΔΔCt^.

### Statistical analysis

All statistical analyses were carried out using the SPSS 18.0 statistical software package (SPSS Inc., Chicago, IL). The two groups were compared using unpaired Student's *t*-test. For statistical analysis, the continuous Trop2 expression data from IHC were first converted into dichotic data (low vs high) using specific cutoff values, which were selected based on significant differences in overall survival (OS) using the X-tile software program (The Rimm Lab at Yale University; http://www.tissuearray.org/rimmlab) [35]. Student's *t*-test and χ^2^ test (T < 1 or *n* < 40, Fisher^’^s exact test) were used to determine the statistical significance of differences between the groups. Cumulative patient survival was estimated using the Kaplan-Meier method, and a log-rank test was used to compare the survival curves. A Cox proportional hazards model was used to calculate univariate and multivariate hazard ratios for the variables. Values of *p* less than 0.05 were considered statistically significant.

## SUPPLEMENTARY FILES


